# Metabolomics Profiles Alterations in Cigarette Smokers and Heated Tobacco Product Users

**DOI:** 10.2188/jea.JE20230170

**Published:** 2024-09-05

**Authors:** Sei Harada, Hideki Ohmomo, Minako Matsumoto, Mizuki Sata, Miho Iida, Aya Hirata, Naoko Miyagawa, Kazuyo Kuwabara, Suzuka Kato, Ryota Toki, Shun Edagawa, Daisuke Sugiyama, Asako Sato, Akiyoshi Hirayama, Masahiro Sugimoto, Tomoyoshi Soga, Masaru Tomita, Atsushi Shimizu, Tomonori Okamura, Toru Takebayashi

**Affiliations:** 1 Department of Preventive Medicine and Public Health, Keio University School of Medicine, Tokyo, Japan; 2 Institute for Advanced Biosciences, Keio University, Yamagata, Japan; 3 Iwate Tohoku Medical Megabank Organization, Iwate Medical University, Iwate, Japan

**Keywords:** heated tobacco products, electronic nicotine delivery devices, metabolomics, prevention, smoking-induced disease

## Abstract

**Background:**

Heated tobacco products (HTPs) have gained global popularity, but their health risks remain unclear. Therefore, the current study aimed to identify plasma metabolites associated with smoking and HTP use in a large Japanese population to improve health risk assessment.

**Methods:**

Metabolomics data from 9,922 baseline participants of the Tsuruoka Metabolomics Cohort Study (TMCS) were analyzed to determine the association between smoking habits and plasma metabolites. Moreover, alterations in smoking-related metabolites among HTP users were examined based on data obtained from 3,334 participants involved from April 2018 to June 2019 in a follow-up survey.

**Results:**

Our study revealed that cigarette smokers had metabolomics profiles distinct from never smokers, with 22 polar metabolites identified as candidate biomarkers for smoking. These biomarker profiles of HTP users were closer to those of cigarette smokers than those of never smokers. The concentration of glutamate was higher in cigarette smokers, and biomarkers involved in glutamate metabolism were also associated with cigarette smoking and HTP use. Network pathway analysis showed that smoking was associated with the glutamate pathway, which could lead to endothelial dysfunction and atherosclerosis of the vessels.

**Conclusion:**

Our study showed that the glutamate pathway is affected by habitual smoking. These changes in the glutamate pathway may partly explain the mechanism by which cigarette smoking causes cardiovascular disease. HTP use was also associated with glutamate metabolism, indicating that HTP use may contribute to the development of cardiovascular disease through mechanisms similar to those in cigarette use.

## INTRODUCTION

In recent years, heated tobacco products (HTPs) have grown in popularity worldwide, including Asia, North America, and Europe.^1^^–^^4^ According to the 2018 National Health and Nutrition Survey,^5^ in Japan, around 30.6% of male habitual smokers and 23.6% of female habitual smokers used HTPs. The estimated prevalence of HTP usage among individuals over the age of 20 years was 8.8% for men and 1.9% for women, and recent data indicates a further upward trend since then.^3^ While HTP companies claim that HTPs are less harmful to health than cigarettes, available evidence has been insufficient. In 2020, the United States Food and Drug Administration granted authorization for the IQOS Tobacco Heating System as modified risk tobacco products, specifically for “exposure modification.” However, the FDA did not issue a “risk modification” order owing to the lack of sufficient evidence, even after reviewing scientific data, public comments, and recommendations from the Tobacco Products Scientific Advisory Committee.^6^ Although the amount of some carcinogens in HTP aerosol ingredients has been significantly reduced, other substances, such as nicotine, have not been reduced as much. Considering that only a few studies have directly examined the adverse health effects of HTPs, the health risks of HTPs have yet to be fully understood to date.

To properly regulate HTPs for public health, quantitative assessment of health risks is necessary. However, just as it required a considerable amount of time to elucidate the effects of cigarettes on cancer, pulmonary diseases, or cardiovascular diseases, establishing evidence of health effects through epidemiological studies would likely to require long-term follow-up studies of a large number of HTP users. Therefore, users will be exposed to HTPs even before any definitive evidence can be established. Regardless of whether HTP use carries a lower disease risk than does cigarette use, if it promotes higher risk for disease than does not smoking cigarettes, it would still be associated with greater health impairment. Therefore, there is an urgent need for more rapid methods of assessing health risks.

The concept of “Meet-in-the-Middle” has been proposed as a method of assessing the risk of rare diseases or those that require long-term follow-up.^7^^–^^9^ This concept aims to assess these diseases at an earlier stage using omics analysis methods, which have been advancing rapidly in recent years. Researchers believe that capturing changes in the body, which occur between exposure and disease onset, through omics analysis can be useful for risk assessment. We have applied this concept to HTP use and shown that DNA methylation and gene expression analysis may be useful to address the risk assessment of HTP use and cancer.^10^

The current study focused on metabolomics among the omics analysis methods. Regarding smoking and metabolome analysis, studies have identified several metabolites affected by smoking.^11^^–^^13^ These may reflect the metabolic changes caused by smoking and could be promising biomarkers that could identify intermediate changes between exposure and disease onset. Specifically, there have been observations associating blood amino acid levels, including glutamate, with cardiovascular diseases.^13^^–^^15^ However, there have not been any metabolomics studies conducted on the Japanese population in relation to tobacco smoking. In addition, metabolomics studies on HTP use have been lacking worldwide.

The present study has two aims: to identify plasma metabolites associated with combustible cigarette smoking in a large Japanese population using metabolomic analysis at baseline when HTPs had not been introduced into the Japanese market (Aim 1) and to determine the levels of these smoking-related metabolites in HTP users and their role in disease risk assessment using 2018–2019 follow-up survey data (Aim 2).

## METHODS

### Study participants

This study was based on the Tsuruoka Metabolomics Cohort Study (TMCS), a prospective cohort study conducted in Tsuruoka City (Yamagata Prefecture, Japan). The baseline survey of the aforementioned study was conducted from fiscal year (April–March) 2012–2015 and enrolled 11,002 participants aged 35–74 years. Details regarding the cohort study are available elsewhere.^16^^–^^18^ The baseline survey of the TMCS was used in the analyses of Aim 1. For Aim 2, we analyzed 3,334 participants involved from April 2018 to June 2019 in a follow-up survey of the TMCS conducted from fiscal year 2018–2021.

The study was approved by the Medical Ethics Committee of the Keio University School of Medicine, Tokyo, Japan (Approval Nos. 20110264 and 20180336). All individual participants in this study provided written informed consent.

### Data and sample collection

All data and samples were obtained during the annual health check-up of the TMCS baseline survey from April 2012 to March 2015 for Aim 1 and the follow-up survey from April 2018 to June 2019 for Aim 2. Information on lifestyle, such as smoking habits, education, medical history, and medications, was collected through a standardized self-administered questionnaire and face-to-face interviews by trained interviewers during the baseline and follow-up surveys. In terms of smoking habits, we interviewed participants about their current smoking status, the number of cigarettes per day, and the age at which they started and quit smoking. Information on HTPs and e-cigarette smoking was also collected at the follow-up survey. Health examination results, including blood pressure, triglycerides, cholesterols, and hemoglobin A1c (HbA1c), were also collected.

Blood samples were collected between 8:30 and 10:30 AM after an overnight fast to avoid variations due to fasting and circadian rhythm. Plasma samples were collected with ethylenediaminetetraacetic acid-2Na as an anticoagulant and kept at 4°C immediately after collection. The samples were centrifuged (1,500 *g* at 4°C for 15 min) within 1 hour of collection, divided into aliquots, and kept for a maximum of 6 hours at 4°C until extraction of metabolites.

For Aim 1, current smokers were defined as those who were smoking at the time of the 2012–2015 baseline survey, past smokers as those who had a smoking habit before the baseline, and never smokers as those who had no smoking habit by the baseline.

For Aim 2, we categorized participants’ smoking habits according to the 2018–2019 follow-up survey. In the follow-up survey, the number of combustible cigarettes and HTPs smoked daily were collected according to product type (IQOS, Philip Morris Inter National Inc., Richmond, VA, USA; glo, British American Tobacco Plc., London, UK; and Ploom TECH, Japan Tobacco Inc., Tokyo, Japan). The tobacco-containing insert of IQOS and glo is a stick, whereas that of Ploom TECH is a capsule. We defined smoking one combustible cigarette as equivalent to smoking one IQOS or glo stick or smoking a Ploom TECH device for a maximum of 10 min. The specific product used was checked by the interviewer using photographs of each HTP type. Participants were categorized into five groups according to smoking habits determined from the follow-up survey: cigarette smokers, HTP users, past smokers, never smokers, and dual smokers who were smoking cigarettes and HTPs. No participant used electric nicotine delivery systems other than HTPs, such as e-cigarettes. Cigarette smokers were identified as smokers who smoked at least one combustible cigarette per day but no HTPs, whereas HTP users were identified as smokers who smoked HTPs at least once per day but no combustible cigarettes. Dual smokers were excluded from the study owing to the small sample size. All smokers at follow-up had started smoking before baseline; accordingly, HTP users had smoked only combustible cigarettes at baseline. Never smokers were defined as those who had never smoked daily, whereas past smokers were defined as those who did not have a smoking habit at the time of the survey but had smoked daily in the past.

### Metabolomics measurement

Plasma samples were collected from 10,993 participants in the baseline survey and 3,334 participants in the follow-up survey underwent metabolomic profiling. Non-targeted mass spectrometry-based metabolomic profiling was performed with fasting plasma samples via capillary electrophoresis time-of-flight mass spectrometry. Metabolite extraction from plasma was completed within 6 hours after collection to minimize metabolic changes in plasma. The extraction method has been described in detail elsewhere.^19^ Capillary electrophoresis time-of-flight mass spectrometry analysis of cationic and anionic metabolites was performed as described previously.^20^^,^^21^ Raw data were processed using our proprietary software (MasterHands).^21^^,^^22^ We used two capillary electrophoresis-mass spectrometry (CE-MS) instruments to measure cations and two to measure anions. During the study period, these four instruments were solely used for this study. Mass calibration using tuning solution and MS entrance cleaning were performed at the beginning of every sequence to ensure robust performance. In addition, to avoid unexpected changes in the sensitivity or variance in the measurement of mass in a continuous run, the number of samples per run was limited to 100. As a preliminary study, we identified 290 metabolite peaks (131 cations and 159 anions) in plasma: 154 known with standard compounds and 136 unknown. We decided a priori to measure the absolute concentrations of 94 metabolites (54 cations and 40 anions) that were expected to be observed stably in most human plasma samples and matched with standard compounds. To monitor the stability of the metabolomics analysis, quality control (QC) samples were injected into every 10 samples and assessed at the start of the analytical run and at various intervals throughout the analysis. For QC samples, 150 mL serum collected from 20 people from the same population in advance was extracted for metabolomics analysis as soon as collected, then divided into 50 µL aliquots and stored at −80°C. QC aliquots stored at −80°C were thawed and used for monitoring daily during the study.^16^

### Statistical analysis

For Aim 1, we used metabolomics data from 9,922 participants included in the baseline survey when HTPs had not been introduced into the Japanese market (4,576 men and 5,346 women), stratified according to sex, to analyze the association between smoking habits and metabolites. To minimize errors due to measurement batches of metabolites, a mixed-model analysis was performed. Accordingly, the measurement batch of the metabolites was considered the random effect, the concentration of each of the 94 metabolites (log-transformed) was considered the object variable, and smoke habit (never smokers [reference], past smokers, and cigarette smokers) was considered the explanatory variable (a fixed effect). Model 1 adjusted for age, whereas model 2 adjusted for age, systolic blood pressure, body mass index (BMI), HbA1c, triglycerides, alcohol consumption, coffee consumption, and education as covariates (fixed effects). To visualize differences in metabolite concentrations across groups, a heat map for smoking biomarkers common to both men and women was generated. Hierarchical clustering was performed for each group and metabolite using Ward’s methods and Euclidean distance.

Next, to examine the dose-response relationship, we examined the relationship between the number of cigarettes smoked and the metabolome for 1,335 male cigarette smokers. Mixed-model analysis was conducted, with the measurement batch of metabolites as the random effect, the concentration of each of the 94 metabolites (log-transformed) as the objective variable, and the number of cigarettes smoked per day as the explanatory variable (a fixed effect). As covariates (fixed effects), the same covariates as in (a) were used.

To examine changes in metabolite concentrations due to smoking cessation, we examined the association between years since smoking cessation and metabolites in 2,287 previous male smokers. Mixed-model analysis was conducted, with the measurement batch of metabolites as the random effect, the concentration of each of the 94 metabolites (log-transformed) as the objective variable, and the period since quitting smoking as the explanatory variable (a fixed effect). In model 1, the same covariates (fixed effects) as in the analysis of the association of smoking habits with metabolites were used. *P* values obtained in analyses were adjusted for multiple comparisons using the false discovery rate (FDR). Finally, we performed pathway network analysis to compare the metabolic pathways between current cigarette smokers and never smokers using Ingenuity Pathway Analysis (Qiagen NV, Venlo, Netherlands; www.qiagen.com/ingenuity).

For Aim 2, to match factors that may affect the metabolomic profiles between HTP users and other groups, each HTP user was matched with two or three participants of each group (never smokers, cigarette smokers, and past smokers). Sex was matched completely, whereas age and drinking habits were matched as much as possible with the HTP users first and then with the following additional conditions.

For never smokers, BMI, systolic blood pressure (SBP), non-high-density lipoprotein (non-HDL), hemoglobin A1c (HbA1c), hypertension medication, dyslipidemia medication, and diabetes treatment were used as factors for propensity score matching. For cigarette smokers, the number of cigarettes smoked per day was used as a factor for propensity score matching. For past smokers, the number of years since quitting smoking was matched to the number of years since switching to HTP.

A mixed-model analysis was then conducted to determine the relationship between smoking habits (HTP users, cigarette smokers, past smokers, and never smokers) and each metabolite identified as being associated with smoking based on the analyses of Aim 1. The measurement batch of the metabolite was considered the random effect; the concentration of each metabolite (log-transformed) was considered the object variable; and smoking habit (never smokers, past smokers, cigarette smokers, and HTP users), age, and sex were considered the explanatory variables (fixed effects). We included age, sex, drinking status, BMI, SBP, HbA1c, and non-HDL as covariates in the fully-adjusted model. A *P*-value was presented as the result of the analysis of variance among the four groups (never smokers, past smokers, cigarette smokers, and HTP users). We conducted a post-hoc analysis to estimate β and its 95% confidence interval between each two groups and show the fold change and its 95% confidence interval for all combinations. We also generated a heat map and performed hierarchical clustering to visualize metabolomic differences according to smoking habits.

Statistical analyses were performed using R.3.6.2 (R Foundation for Statistical Computing, Vienna, Austria). We used MetaboAnalyst 5.0 (https://www.metaboanalyst.ca/) to generate heat maps and conduct hierarchical clustering.

## RESULTS

### Characteristics

Table 1 shows the characteristics of the three groups of participants divided according to smoking habits at baseline. Men had a much higher smoking prevalence and number of cigarettes smoked than did women. Current combustible cigarette smokers and past smokers tended to have a higher drinking prevalence than never smokers. Coffee consumption was higher among current male smokers and current and past female smokers than among never smokers. Among women, current cigarette smokers were younger; had lower blood pressure; had a lower prevalence of hypertension, diabetes, and dyslipidemia; and were more educated than never smokers. Among men, current cigarette smokers tended to have lower blood pressure than never smokers, although not as pronounced as that among women. No marked differences between groups were observed in BMI, HbA1c or cholesterols.

**Table 1.** Characteristics of the participants during the baseline survey

	Men (4,576)			Women (5,346)		
	Current cigarette smokers	Past smokers	Never smokers	Current cigarette smokers	Past Smokers	Never smokers
*N*, %	1,335 (29.2)	2,287 (50.0)	954 (20.8)	238 (4.5)	471 (8.8)	4,637 (86.7)
Age, mean (SD)	56.64 (9.77)	61.13 (8.93)	58.83 (11.44)	49.84 (9.55)	52.95 (10.56)	60.06 (9.86)
Number of smoking, mean (SD)	18.07 (7.26)	20.67 (9.91)	—	10.74 (5.06)	10.40 (7.34)	—
Years of smoking, mean (SD)	35.58 (10.26)	22.97 (12.48)	—	25.06 (9.38)	14.61 (9.81)	—
Pack-year, mean (SD)	648.97 (324.38)	500.83 (384.59)	—	283.86 (190.91)	171.59 (198.67)	—
<200, %	77 (5.8)	423 (21.3)	—	90 (38.0)	296 (69.0)	—
200–399, %	209 (15.7)	457 (23.0)	—	93 (39.2)	82 (19.1)	—
400–600, %	298 (22.4)	383 (19.3)	—	31 (13.1)	25 (5.8)	—
>600, %	749 (56.2)	725 (36.5)	—	23 (9.7)	26 (6.1)	—
Years after cessation, mean (SD)	—	16.15 (11.79)	—	—	12.41 (9.74)	—
10, %	—	594 (34.5)	—	—	161 (42.4)	—
10–20, %	—	473 (27.4)	—	—	125 (32.9)	—
>20, %	—	657 (38.1)	—	—	94 (24.7)	—
Body mass index, mean (SD)	23.31 (3.20)	24.12 (2.97)	23.89 (2.98)	22.57 (4.05)	22.93 (3.79)	22.80 (3.41)
Systolic blood pressure, mean (SD)	128.56 (18.19)	133.36 (17.25)	131.46 (16.79)	117.94 (17.24)	123.16 (18.49)	126.71 (18.82)
Diastolic blood pressure, mean (SD)	77.72 (11.37)	80.83 (10.58)	79.73 (10.88)	68.62 (11.47)	72.41 (11.36)	72.88 (10.90)
Hypertension medication, %	285 (21.3)	826 (36.1)	275 (28.8)	16 (6.7)	77 (16.3)	1203 (25.9)
HbA1c, mean (SD)	5.73 (0.70)	5.76 (0.64)	5.68 (0.66)	5.51 (0.47)	5.59 (0.51)	5.66 (0.46)
Diabetes medication, %	97 (7.3)	251 (11.0)	65 (6.8)	6 (2.5)	13 (2.8)	205 (4.4)
LDL-cholesterol, median (IQR)	112.6 (92.7–132.0)	115.2 (95.4–134.6)	116.2 (98.4–135.6)	108.4 (88.0–132.2)	113.3 (92.4–137.0)	118.4 (99.4–139.2)
HDL-cholesterol, median (IQR)	57.0 (48.0–68.0)	61.0 (51.0–72.0)	61.0 (52.0–71.0)	66.5 (57.0–80.0)	71.0 (61.0–84.0)	71.0 (60.0–82.0)
Triglycerides, median (IQR)	116.0 (82.5–176.0)	105.0 (75.0–154.0)	95.0 (67.0–131.0)	85.0 (61.3–110.0)	78.0 (57.5–109.0)	79.0 (59.0–107.0)
Dyslidipemia medication, %	111 (8.3)	405 (17.7)	126 (13.2)	18 (7.6)	54 (11.5)	1,078 (23.2)
Daily drinker, %	840 (63.0)	1,408 (61.6)	397 (41.7)	66 (27.8)	115 (24.4)	269 (5.8)
Coffee >1 cup, %	976 (74.1)	1,347 (59.5)	544 (57.6)	204 (86.1)	367 (88.8)	3,167 (69.3)
Education						
9–12 years, %	175 (13.2)	342 (15.0)	145 (15.3)	15 (6.4)	38 (8.1)	752 (16.3)
12–16 years, %	787 (59.3)	1,281 (56.2)	492 (51.9)	116 (49.2)	197 (41.9)	2,443 (52.9)
>16 years, %	366 (27.6)	658 (28.8)	311 (32.8)	105 (44.5)	235 (50.0)	1,427 (30.9)

IQR, interquartile range; SD, standard deviation.

### Association between smoking and plasma metabolome

**Figure 1.**
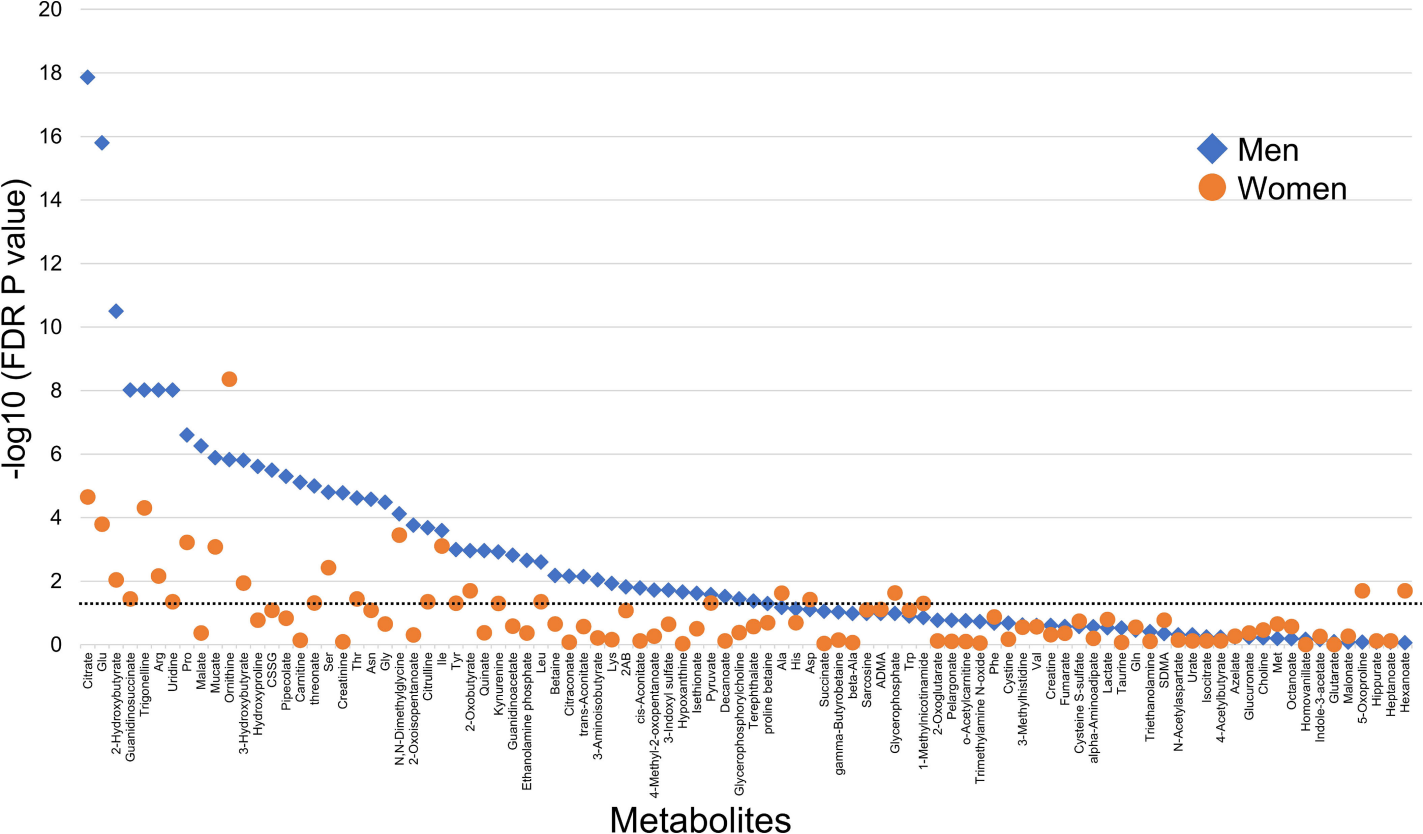
Metabolites associated with habitual smoking. Each plot displays the *P*-value derived from a mixed-model analysis, where the dependent variable (fixed effect) is the concentration of 1 of the 94 metabolites (log-transformed). The independent variable (fixed effect) represents smoking habits (never smokers [reference] and current cigarette smokers). Covariates (fixed effects) account for age, systolic blood pressure, body mass index, HbA1c, triglycerides, and alcohol and coffee consumption. The random effect pertains to the measurement batch in metabolomics analysis. The horizontal line indicates a false discovery rate *P*-value = 0.05. FDR, false discovery rate.

After adjusting for confounders, 48 polar metabolites in men and 28 metabolites in women were found to be associated with cigarette smoking (FDR, *P* < 0.05) (Figure 1 and eTable 1). Moreover, 22 metabolites were commonly associated with smoking in both sexes and were subsequently identified as candidate smoking-related biomarkers (eTable 1).

**Figure 2.**
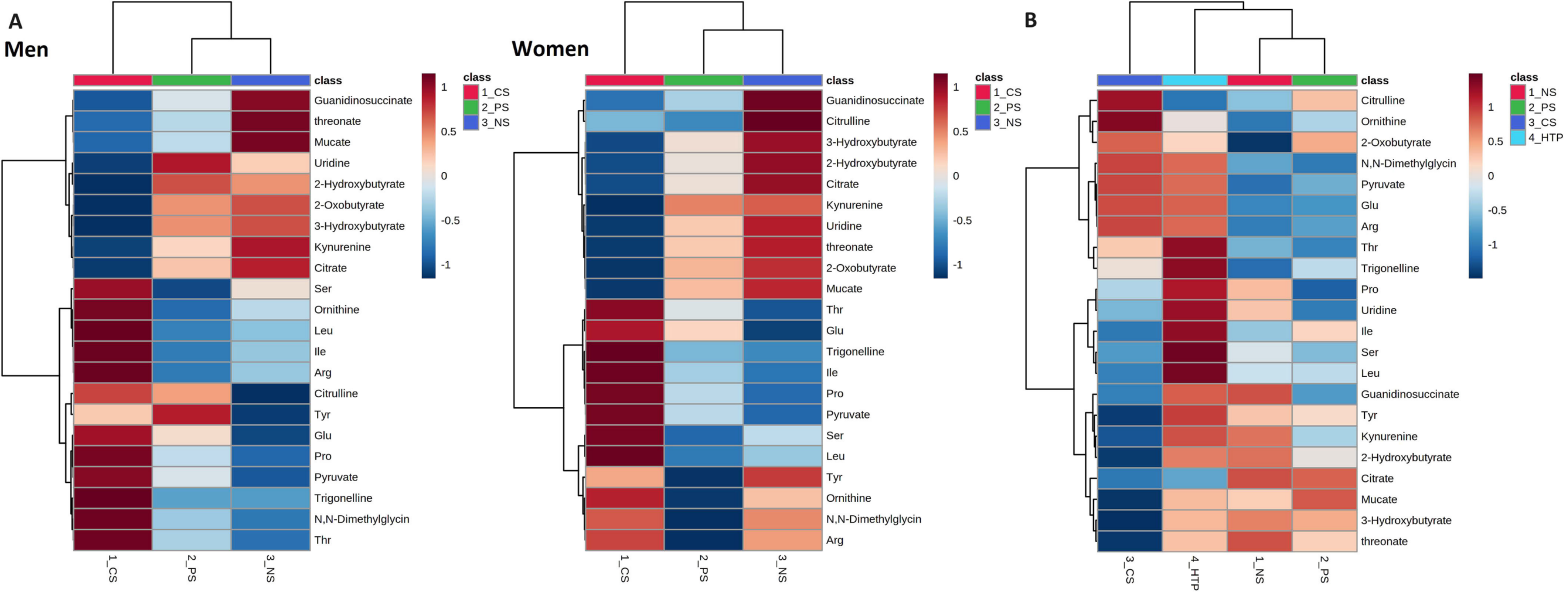
Heat maps of metabolites by habitual smoking groups. (**A**) Heat maps display smoking-related metabolites by habitual smoking groups (current cigarette smokers [CS], past smokers [PS], and never smokers [NS]) at the baseline survey, separate for each sex. Hierarchical clustering was applied to group both metabolites and individuals using Ward’s methods and Euclidean distance. (**B**) Heat map illustrates smoking-related metabolites by habitual smoking groups (cigarette smokers, HTP users, past smokers, and never smokers) during the follow-up survey. Similar to the previous analysis, hierarchical clustering was employed for grouping metabolites and participants using Ward’s methods and Euclidean distance.

Metabolomics differences among the smoking groups are shown in the hierarchical clustering heatmaps (Figure 2A). Current cigarette smokers had higher concentrations of broad amino acid metabolites (including glutamate, proline, and ornithine) and some other compounds (such as trigonelline) than other groups. Current cigarette smokers had lower concentrations of organic acids (including citrates and 2-hydroxybutyrate) and some other compounds (such as guanidinosuccinate and mucate) than other groups. Overall, the metabolomics profile of past smokers was closer to that of never smokers than current cigarette smokers. The metabolite concentrations of past smokers were generally between that of never smokers and current cigarette smokers.

**Figure 3.**
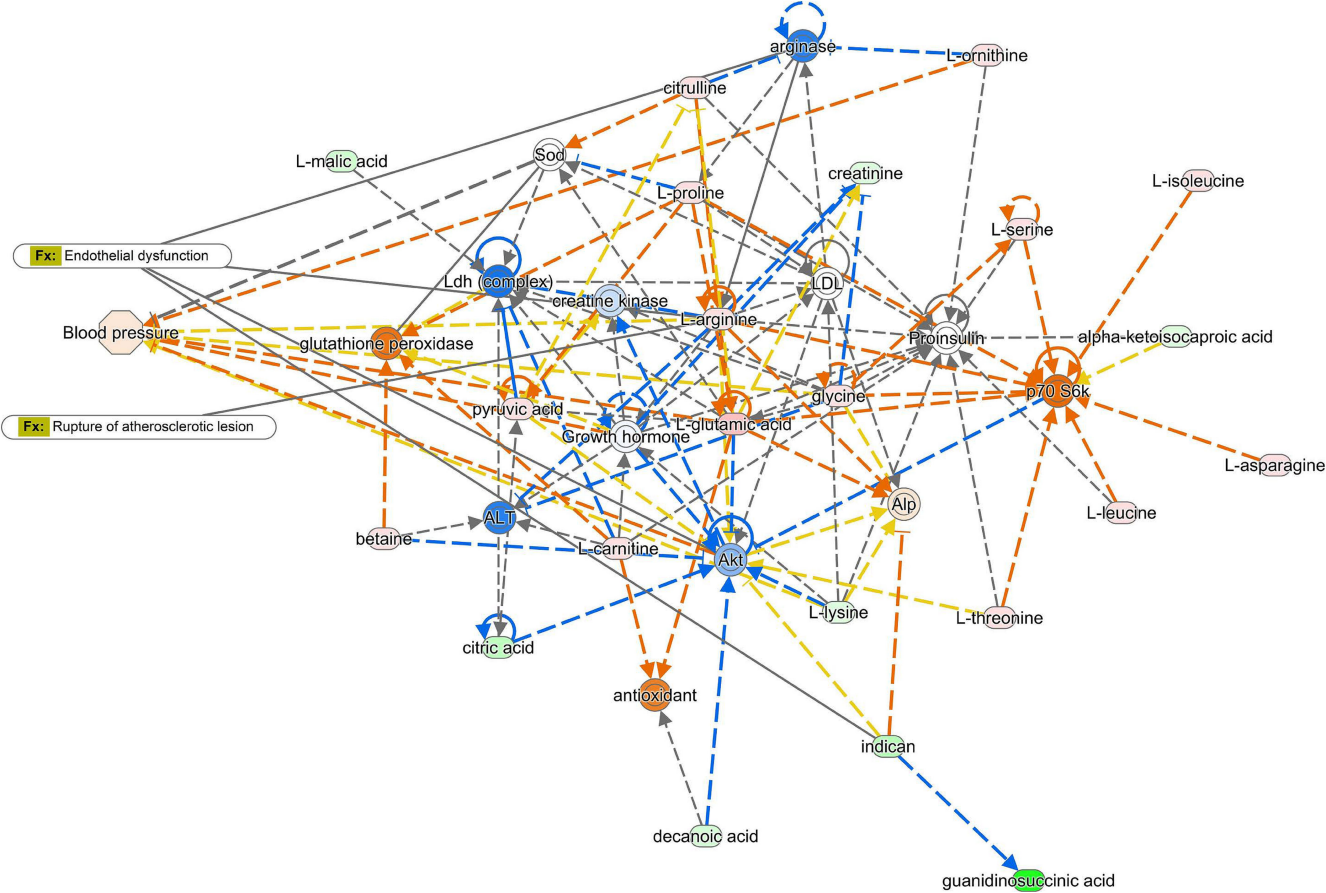
Pathway analysis comparing the metabolic pathways between current cigarette smokers and never smokers. We conducted a pathway network analysis using Ingenuity Pathway Analysis (Qiagen NV, Venlo, Netherlands; www.qiagen.com/ingenuity) to compare metabolic pathways between current cigarette smokers and never smokers. In the figure, the metabolites shown in red and green represent higher and lower abundance levels in current cigarette smokers, respectively. Similarly, the proteins in orange and blue indicate higher and lower estimated abundance levels in current cigarette smokers. This analysis revealed a connection between smoking and the glutamate pathway, which has the potential to contribute to the development of atherosclerosis and endothelial dysfunction in blood vessels.

Several metabolites (glutamate, arginine, ornithine, and citrulline) that showed strong positive associations with current cigarette smokers especially in men belonged to the glutamate metabolic pathway. Network pathway analysis using Ingenuity Pathway Analysis showed that smoking was associated with the glutamate pathway, which could lead to atherosclerosis and endothelial dysfunction of the vessels (Figure 3).

### Dose-response relationship between the number of cigarettes and plasma metabolome

We examined the dose–response relationship between current cigarette smoking and the metabolome in male cigarette smokers. Among the 22 candidate smoking-related biomarkers, glutamate and trigonelline were still associated with the number of cigarettes smoked after multivariable adjustment (*P* = 0.005 after FDR adjustment for both).

### Association between years since smoking cessation and plasma metabolome

Among the 22 candidate smoking-related biomarkers, glutamate (FDR *P* = 3.9 × 10^−6^) and mucic acid (FDR *P* = 0.013) remained associated with the number of years since smoking cessation after multivariable adjustment.

### Association between HTPs and plasma metabolome

The characteristics of the participants after matching are shown in eTable 2. All three groups had similar characteristics except that cigarette smokers were slightly older.

Metabolomic differences between HTP users and other smoking groups are shown in the hierarchical clustering heatmaps (Figure 2B). The result of hierarchical clustering suggested that the metabolomic profile of HTP users was between cigarette smokers and never smokers (never smokers), albeit closer to cigarette smokers. In particular, cigarette smokers and HTP users had higher concentrations of trigonelline and amino acid metabolites, including glutamate, ornithine, and arginine, than did never smokers and past smokers.

We then examined the association between smoking habits and biomarkers of glutamate metabolism (glutamate, arginine, ornithine, and citrulline) and trigonelline, which have a clear association with cigarette smoking (Table 2). A comparison of the four groups showed apparent group differences in glutamate and trigonelline and marginal group differences in ornithine. In comparison between HTP users and cigarette smokers, minimal differences were detected for these five metabolites, confirming the similarity between HTP users and cigarette smokers in terms of the concentrations of glutamate-related metabolites and trigonelline. After adjusting for confounding factors, HTP users showed apparent differences in glutamate and trigonelline and marginal differences in ornithine compared to never smokers. This trend was similar to the results obtained when comparing smokers with never smokers. Past smokers were similar to never smokers in glutamate-related metabolite concentrations, but the trigonelline level in past smokers was slightly higher than that of never smokers.

**Table 2.** Metabolic changes in smoking habits including heated tobacco product use

	4-group comparison	HTP vs NS	HTP vs PS	HTP vs CS	CS vs NS	CS vs PS	PS vs NS

Age- and sex-adjusted	*P*-value	fold change (95% confidence interval)					
Glutamate	0.014	1.43 (1.05–1.95)	1.45 (1.06–1.98)	1.10 (0.80–1.53)	1.30 (1.01–1.66)	1.31 (1.03–1.68)	0.99 (0.78–1.24)
Arginine	0.726	1.19 (0.87–1.64)	1.18 (0.86–1.63)	1.03 (0.74–1.44)	1.15 (0.89–1.49)	1.14 (0.89–1.47)	1.01 (0.79–1.28)
Ornithine	0.083	1.25 (0.91–1.71)	1.14 (0.84–1.56)	0.88 (0.64–1.22)	1.41 (1.10–1.81)	1.30 (1.01–1.66)	1.09 (0.87–1.38)
Citrulline	0.572	0.97 (0.71–1.33)	0.90 (0.66–1.23)	0.83 (0.60–1.15)	1.17 (0.91–1.51)	1.08 (0.85–1.39)	1.08 (0.86–1.37)
Trigonelline	0.0004	1.65 (1.27–2.14)	1.29 (1.00–1.68)	1.04 (0.79–1.36)	1.59 (1.28–1.98)	1.25 (1.01–1.55)	1.27 (1.04–1.56)
Fully-adjusted^*^							
Glutamate	0.00003	1.40 (1.08–1.82)	1.42 (1.09–1.84)	1.02 (0.78–1.33)	1.38 (1.13–1.69)	1.40 (1.14–1.71)	0.99 (0.82–1.20)
Arginine	0.616	1.23 (0.89–1.70)	1.20 (0.87–1.65)	1.03 (0.74–1.44)	1.19 (0.93–1.53)	1.16 (0.91–1.49)	1.03 (0.81–1.30)
Ornithine	0.081	1.32 (0.96–1.82)	1.17 (0.85–1.61)	0.92 (0.66–1.29)	1.43 (1.11–1.83)	1.27 (0.99–1.62)	1.13 (0.89–1.42)
Citrulline	0.529	0.93 (0.67–1.29)	0.85 (0.61–1.18)	0.80 (0.57–1.12)	1.17 (0.91–1.51)	1.07 (0.83–1.37)	1.10 (0.86–1.39)
Trigonelline	0.005	1.60 (1.22–2.10)	1.27 (0.97–1.66)	1.02 (0.77–1.35)	1.57 (1.25–1.97)	1.25 (1.00–1.55)	1.26 (1.02–1.55)

CS, current cigarette smokers; HTP, heated tobacco product users; NS, never smokers; PS, past smokers.

^*^We included age, sex, drinking status, body mass index, systolic blood pressure, HbA1c, and non-high-density-lipoprotein cholesterol as covariates in the fully-adjusted model.

## DISCUSSION

Our large-scale plasma metabolomic profiling of 9,922 Japanese participants showed that combustible cigarette smokers and never smokers had very different metabolic characteristics and identified 22 polar metabolites that could be candidate biomarkers for smoking. We also showed that HTP users had metabolite profiles closer to cigarette smokers than nonsmokers, including glutamate metabolism and trigonelline.

The level of plasma glutamate was higher in cigarette smokers than in never smokers. Glutamate levels tended to be higher in men who smoked more and lower in participants who had quit smoking for an extended period. These results suggest that glutamate may be a useful biomarker for smoking. The association between smoking and smoking cessation and glutamate observed herein was consistent with that reported in a previous cohort study.^13^ Our study had a larger sample size and showed that these findings were also observed in Asians.

Apart from plasma glutamate, other biomarkers included in glutamate metabolism (ie, glutamate, arginine, ornithine, and citrulline) also showed a strong association with smoking. Previous studies have reported that the glutamate transporter encoded by *SLC7A11* was activated in cigarette smokers,^23^^,^^24^ which promoted an increase in circulating glutamate, arginine, ornithine, and citrulline. This mechanism may be responsible for the elevated glutamate metabolism-related biomarkers in cigarette smokers (eFigure 1). Previous studies have suggested that elevated levels of this metabolic pathway can lead to endothelial cell damage through decreased nitric oxide and contribute to atherosclerosis.^25^^–^^27^

Several reports have suggested that glutamate concentration is associated with the future development of cardiovascular diseases^14^^,^^15^ and contributes to plaque development in atherosclerosis.^28^ Changes in the glutamate pathway may partly explain the mechanism of cardiovascular disease caused by smoking. Our pathway network analysis results also support this hypothesis.

In the present study, HTP users and cigarette smokers showed similar changes in glutamate metabolism and trigonelline. HTP users had switched from cigarettes to HTPs due to the recent popularity of these novel products. It is suggested that even after switching to HTPs, the effects of smoking on metabolism remain.

The residual effects on glutamate metabolism may suggest that the effects of HTPs on cardiovascular disease are not significantly different from those of cigarettes. Although the health effects of HTPs are not well understood, nicotine, which is believed to affect cardiovascular disease^29^^,^^30^ by influencing the sympathetic nervous system, promoting atherosclerosis, and inducing endothelial cell injury, is hardly reduced in the aerosol of HTPs.^31^^–^^33^

To our knowledge, our study is the first epidemiologic study to show the metabolomic profile in plasma for HTP users, indicating the underlying mechanisms related to the elevated risk of cardiovascular disease in HTP users. Given that there is currently little evidence on the health effects of HTP, our results may provide valuable insight into the disease risk of HTP use.

One of the strengths of this study is that our CE-MS metabolomics platform is stable and reliable enough for epidemiologic studies. Our previous study showed that measurements of QC samples have been stable for approximately 4 years, with baseline survey samples measured, indicating that the metabolomics measurement in this study is sufficiently reproducible for use in epidemiologic studies. This reproducibility was also observed in the specimens in the follow-up study. Changes in QC sample measurements have been limited, stable, constant, and reproducible from the baseline survey to the present, regardless of the frozen storage period. Our validation study also showed that our CE-MS platform has good coverage and quantification for hydrophilic and ionic metabolites, with equal or better measurement reproducibility compared to other measurement platforms.^16^

One of the limitations of this study is its cross-sectional design. Therefore, our results should be interpreted with caution. The association between cigarette smoking and plasma glutamate may be reliable given that a dose–response relationship and a negative association with years of smoking cessation were observed.

Other limitations for Aim 2 include the short-term observation since switching to HTPs, the small sample size of heated tobacco users, and the incomplete matching of characteristics between the groups. However, HTPs are novel and rapidly gaining in popularity, despite the uncertain risks of HTP use. From a public health perspective, uncertain risks need to be proactively prevented. Considering that it will take too long to fully establish the risks of HTPs, this study, despite its relatively small population and short-term metabolic changes, may contribute to a rapid consideration of preventive measures for HTP use. Further follow-up studies are needed to examine the long-term effects of HTP use and its relationship with cardiovascular disease outcomes. We also expect findings from other populations to accumulate in the near future.

## SUPPLEMENTARY MATERIAL

eTable 1. Metabolic changes in smoking habits

eTable 2. Characteristics of the participants at the follow-up survey after matching

eFigure 1. Glutamate pathway and atherosclerosis development in smokers.
