# Supplementary material for: Metabolomics Profiles Alterations in Cigarette Smokers and Heated Tobacco Product Users
Source: J Epidemiol. 2024 Sep 5;34(9):403–10. doi: 10.2188/jea.JE20230170 (PMC11330708; doi:10.2188/jea.JE20230170)
Supplement: Supplementary file 1 [file je-34-403-s001.pdf]

eTable 1. Metabolic changes in smoking habits

	HMDB_ID	Metabolite name	Men								Women								HMDB_ID	Metabolite name
			Cigarette smokers vs. never smokers				Past smokers vs. never smokers				Cigarette smokers vs. never smokers				Past smokers vs. never smokers					
			Age adjusted		Fully adjusted		Age adjusted		Fully adjusted		Age adjusted		Fully adjusted		Age adjusted		Fully adjusted			
			Fold change (ref: Never)	FDR *P* value	Fold change (ref: Never)	FDR *P* value	Fold change (ref: Never)	FDR *P* value	Fold change (ref: Never)	FDR *P* value	Fold change (ref: Never)	FDR *P* value	Fold change (ref: Never)	FDR *P* value	Fold change (ref: Never)	FDR *P* value	Fold change (ref: Never)	FDR *P* value		
1	HMDB0000094	Citrate	0.89	5.2.E-34	0.92	1.4.E-18	0.96	4.8.E-06	0.98	7.0.E-02	0.90	5.8.E-12	0.93	2.2.E-05	0.97	7.9.E-02	1.00	9.0.E-01	HMDB0000094	Citrate
2	HMDB0000148	Glu	1.17	1.8.E-18	1.14	1.6.E-16	1.11	8.7.E-09	1.05	1.8.E-02	1.19	1.1.E-07	1.12	1.6.E-04	1.13	8.3.E-07	1.06	4.2.E-02	HMDB0000148	Glu
3	HMDB0000008	2-Hydroxybutyrate	0.89	6.1.E-12	0.89	3.1.E-11	1.03	2.0.E-01	1.00	9.4.E-01	0.89	4.6.E-05	0.92	8.9.E-03	0.97	5.3.E-01	0.96	4.4.E-01	HMDB0000008	2-Hydroxybutyrate
4	HMDB0003157	Guanidinosuccinate	0.69	1.2.E-13	0.74	9.6.E-09	0.81	1.0.E-05	0.87	2.6.E-02	0.77	2.4.E-03	0.81	3.6.E-02	0.89	1.2.E-01	0.94	6.0.E-01	HMDB0003157	Guanidinosuccinate
5	HMDB0000875	Trigonelline	1.77	1.2.E-13	1.56	9.6.E-09	1.08	4.3.E-01	1.13	2.9.E-01	2.01	3.7.E-08	1.71	4.9.E-05	1.14	2.9.E-01	1.11	5.9.E-01	HMDB0000875	Trigonelline
6	HMDB0000517	Arg	1.04	1.8.E-07	1.05	9.6.E-09	0.99	1.9.E-01	1.00	8.0.E-01	1.04	8.5.E-03	1.05	6.8.E-03	1.00	8.3.E-01	1.00	9.9.E-01	HMDB0000517	Arg
7	HMDB0000296	Uridine	0.96	3.4.E-10	0.96	9.6.E-09	0.99	4.3.E-01	0.99	3.9.E-01	0.97	9.9.E-03	0.97	4.4.E-02	0.99	4.4.E-01	0.99	7.8.E-01	HMDB0000296	Uridine
8	HMDB0000162	Pro	1.06	1.6.E-06	1.07	2.5.E-07	1.03	5.4.E-02	1.02	2.0.E-01	1.08	2.6.E-04	1.08	5.9.E-04	1.02	2.3.E-01	1.02	5.8.E-01	HMDB0000162	Pro
9	HMDB0000639	Mucate	0.89	6.1.E-12	0.92	1.3.E-06	0.94	2.2.E-04	0.96	8.4.E-02	0.91	2.1.E-06	0.93	8.2.E-04	0.97	3.3.E-01	0.99	9.0.E-01	HMDB0000639	Mucate
10	HMDB0000214	Ornithine	1.03	3.1.E-03	1.05	1.5.E-06	0.98	1.5.E-01	1.00	8.0.E-01	1.11	1.2.E-08	1.11	4.3.E-09	1.01	4.9.E-01	1.01	5.9.E-01	HMDB0000214	Ornithine
11	HMDB0000011	3-Hydroxybutyrate	0.79	9.2.E-15	0.86	1.6.E-06	0.95	1.0.E-01	0.98	7.5.E-01	0.74	8.4.E-08	0.85	1.1.E-02	0.90	7.0.E-02	0.94	7.6.E-01	HMDB0000011	3-Hydroxybutyrate
12	HMDB0000943	threonate	0.93	1.1.E-09	0.94	1.0.E-05	0.95	7.7.E-05	0.97	5.9.E-02	0.95	1.4.E-03	0.96	4.8.E-02	0.99	9.4.E-01	1.01	9.0.E-01	HMDB0000943	threonate
13	HMDB0000187	Ser	1.01	3.1.E-01	1.04	1.6.E-05	0.99	5.8.E-01	1.02	1.8.E-01	1.02	3.8.E-01	1.05	3.7.E-03	0.97	1.8.E-02	0.99	5.9.E-01	HMDB0000187	Ser
14	HMDB0000167	Thr	1.05	5.1.E-08	1.04	2.4.E-05	1.01	1.8.E-01	1.01	5.7.E-01	1.04	1.0.E-02	1.04	3.6.E-02	1.01	4.0.E-01	1.01	7.8.E-01	HMDB0000167	Thr
15	HMDB0000092	N,N-Dimethylglycine	1.05	3.3.E-03	1.07	7.6.E-05	1.00	9.3.E-01	1.01	5.7.E-01	1.09	8.5.E-04	1.10	3.5.E-04	1.01	5.8.E-01	1.02	5.9.E-01	HMDB0000092	N,N-Dimethylglycine
16	HMDB0000904	Citrulline	1.02	1.4.E-02	1.04	2.1.E-04	0.99	5.8.E-01	1.01	5.3.E-01	1.02	2.1.E-01	1.04	4.4.E-02	0.99	4.4.E-01	1.00	9.9.E-01	HMDB0000904	Citrulline
17	HMDB0000172	Ile	1.02	1.4.E-02	1.03	2.5.E-04	1.00	9.3.E-01	1.00	7.2.E-01	1.05	3.7.E-04	1.05	7.7.E-04	1.02	1.8.E-01	1.01	5.9.E-01	HMDB0000172	Ile
18	HMDB0000158	Tyr	1.03	1.5.E-03	1.03	1.0.E-03	1.02	3.6.E-03	1.01	3.0.E-01	1.05	1.3.E-03	1.03	4.9.E-02	1.02	1.1.E-01	1.00	9.6.E-01	HMDB0000158	Tyr
19	HMDB0000005	2-Oxobutyrate	0.75	1.1.E-08	0.83	1.1.E-03	0.93	2.0.E-01	0.98	7.8.E-01	0.70	2.4.E-05	0.81	2.0.E-02	0.95	8.3.E-01	0.99	9.0.E-01	HMDB0000005	2-Oxobutyrate
20	HMDB0000684	Kynurenine	0.93	1.3.E-03	0.93	1.2.E-03	0.98	4.2.E-01	0.97	4.2.E-01	0.94	1.8.E-01	0.91	5.0.E-02	1.00	9.3.E-01	0.99	8.9.E-01	HMDB0000684	Kynurenine
21	HMDB0000687	Leu	1.02	4.0.E-02	1.02	2.5.E-03	1.00	9.3.E-01	1.00	6.5.E-01	1.04	7.2.E-04	1.03	4.4.E-02	1.02	4.1.E-02	1.01	7.8.E-01	HMDB0000687	Leu
22	HMDB0000243	Pyruvate	1.06	3.6.E-04	1.04	2.6.E-02	1.05	1.0.E-02	1.02	5.7.E-01	1.06	2.7.E-02	1.06	4.8.E-02	1.02	6.7.E-01	1.01	9.0.E-01	HMDB0000243	Pyruvate
23	HMDB0000156	Malate	0.95	1.9.E-04	0.94	5.5.E-07	1.01	6.7.E-01	0.99	7.2.E-01	0.96	6.7.E-02	0.98	4.3.E-01	1.01	9.4.E-01	1.01	9.0.E-01	HMDB0000156	Malate
24	HMDB0000725	Hydroxyproline	1.06	2.1.E-05	1.08	2.5.E-06	1.00	9.3.E-01	1.00	8.6.E-01	1.05	6.2.E-02	1.04	1.7.E-01	1.04	8.5.E-02	1.02	5.4.E-01	HMDB0000725	Hydroxyproline
25	HMDB0000656	CSSG	0.80	1.6.E-13	0.87	3.2.E-06	0.86	2.6.E-07	0.94	1.8.E-01	0.89	3.2.E-03	0.93	8.2.E-02	0.89	2.6.E-04	0.94	1.7.E-01	HMDB0000656	CSSG
26	HMDB0000070	Pipecolate	1.18	1.0.E-12	1.11	5.0.E-06	1.07	3.6.E-03	1.03	4.7.E-01	1.11	2.0.E-03	1.06	1.4.E-01	1.08	4.2.E-03	1.04	5.4.E-01	HMDB0000070	Pipecolate
27	HMDB0000062	Carnitine	1.06	1.2.E-13	1.04	7.7.E-06	1.04	2.6.E-07	1.02	3.6.E-02	1.01	6.2.E-01	0.99	7.2.E-01	1.01	5.0.E-01	0.99	7.8.E-01	HMDB0000062	Carnitine
28	HMDB0000562	Creatinine	0.96	5.1.E-08	0.97	1.6.E-05	0.99	2.4.E-01	0.99	4.7.E-01	1.00	8.5.E-01	1.00	8.0.E-01	1.00	8.3.E-01	1.00	8.9.E-01	HMDB0000562	Creatinine
29	HMDB0000168	Asn	1.02	1.0.E-03	1.03	2.6.E-05	1.00	8.0.E-01	1.01	4.7.E-01	1.02	8.7.E-02	1.02	8.2.E-02	0.99	3.0.E-01	0.99	5.8.E-01	HMDB0000168	Asn
30	HMDB0000123	Gly	1.02	6.6.E-03	1.04	3.3.E-05	0.98	1.4.E-02	1.00	7.8.E-01	1.02	5.1.E-01	1.03	2.2.E-01	0.96	1.1.E-02	0.97	1.7.E-01	HMDB0000123	Gly
31	HMDB0000019	2-Oxoisopentanoate	0.97	9.4.E-05	0.97	1.7.E-04	1.01	5.0.E-01	0.99	7.2.E-01	0.98	2.5.E-01	0.99	4.9.E-01	1.00	9.4.E-01	0.99	9.0.E-01	HMDB0000019	2-Oxoisopentanoate
32	HMDB0003072	Quinate	1.37	6.0.E-07	1.24	1.1.E-03	1.11	1.6.E-01	1.11	2.6.E-01	1.43	1.6.E-03	1.13	4.2.E-01	1.16	3.3.E-01	1.07	9.0.E-01	HMDB0003072	Quinate
33	HMDB0000128	Guanidinoacetate	1.05	2.6.E-05	1.04	1.5.E-03	1.02	1.5.E-01	1.01	4.7.E-01	1.02	3.4.E-01	1.02	2.6.E-01	1.01	4.9.E-01	1.02	5.9.E-01	HMDB0000128	Guanidinoacetate
34	HMDB0000224	Ethanolamine phosphate	0.87	1.8.E-03	0.87	2.2.E-03	0.96	3.6.E-01	0.95	5.7.E-01	0.92	2.6.E-01	0.93	4.3.E-01	0.96	8.3.E-01	0.98	9.0.E-01	HMDB0000224	Ethanolamine phosphate
35	HMDB0000043	Betaine	1.03	9.0.E-03	1.03	6.6.E-03	1.01	5.5.E-01	1.02	3.0.E-01	1.01	6.0.E-01	1.03	2.2.E-01	0.99	4.6.E-01	1.00	8.9.E-01	HMDB0000043	Betaine
36	HMDB0000634	Citraconate	1.23	4.4.E-04	1.19	6.9.E-03	1.05	4.5.E-01	1.06	5.7.E-01	1.01	9.4.E-01	1.03	8.2.E-01	0.99	9.4.E-01	0.99	9.4.E-01	HMDB0000634	Citraconate
37	HMDB0000958	trans-Aconitate	0.89	5.5.E-03	0.89	7.1.E-03	0.97	4.5.E-01	0.96	5.7.E-01	1.13	8.7.E-02	1.10	2.6.E-01	1.01	9.4.E-01	1.00	9.4.E-01	HMDB0000958	trans-Aconitate
38	HMDB0003911	3-Aminoisobutyrate	0.86	2.2.E-04	0.89	9.1.E-03	0.95	3.2.E-01	0.97	6.5.E-01	0.97	7.5.E-01	1.04	6.1.E-01	0.96	4.9.E-01	1.00	9.9.E-01	HMDB0003911	3-Aminoisobutyrate
39	HMDB0000182	Lys	0.98	1.0.E-03	0.98	1.2.E-02	0.98	3.7.E-02	0.99	2.0.E-01	1.00	7.5.E-01	0.99	6.9.E-01	1.01	5.5.E-01	1.00	9.9.E-01	HMDB0000182	Lys
40	HMDB0000650	2AB	0.96	1.5.E-03	0.97	1.5.E-02	1.02	4.0.E-01	1.00	8.0.E-01	0.95	3.9.E-02	0.96	8.3.E-02	0.99	5.8.E-01	0.97	1.7.E-01	HMDB0000650	2AB
41	HMDB0000072	cis-Aconitate	0.97	5.5.E-03	0.97	1.6.E-02	1.02	2.0.E-01	1.01	7.2.E-01	0.97	1.9.E-01	0.99	7.6.E-01	1.00	9.9.E-01	1.00	9.0.E-01	HMDB0000072	cis-Aconitate
42	HMDB0000695	4-Methyl-2-oxopentanoate	0.98	1.3.E-01	0.98	1.9.E-02	1.01	2.3.E-01	0.99	7.2.E-01	1.02	1.9.E-01	1.01	5.3.E-01	1.01	9.4.E-01	0.99	9.0.E-01	HMDB0000695	4-Methyl-2-oxopentanoate
43	HMDB0000682	3-Indoxyl sulfate	0.89	1.9.E-03	0.91	1.9.E-02	0.94	1.0.E-01	0.95	5.7.E-01	0.92	2.0.E-01	0.91	2.2.E-01	1.03	9.4.E-01	1.03	9.0.E-01	HMDB0000682	3-Indoxyl sulfate
44	HMDB0000157	Hypoxanthine	1.05	3.4.E-01	1.12	2.2.E-02	1.03	5.8.E-01	1.05	4.7.E-01	0.98	7.8.E-01	1.01	9.2.E-01	0.99	9.1.E-01	1.00	9.9.E-01	HMDB0000157	Hypoxanthine
45	HMDB0003903	Isethionate	0.97	4.7.E-02	0.96	2.4.E-02	0.99	7.1.E-01	1.00	9.2.E-01	0.97	4.0.E-01	0.96	3.1.E-01	1.00	9.9.E-01	1.00	9.4.E-01	HMDB0003903	Isethionate
46	HMDB0000511	Decanoate	0.95	2.6.E-02	0.94	3.0.E-02	0.99	6.8.E-01	0.99	7.5.E-01	0.98	6.6.E-01	0.98	7.6.E-01	0.99	9.4.E-01	0.99	9.0.E-01	HMDB0000511	Decanoate
47	HMDB0000086	Glycerophosphorylcholine	0.98	5.9.E-01	0.94	3.6.E-02	0.99	7.1.E-01	0.97	4.7.E-01	0.98	7.2.E-01	0.96	4.1.E-01	0.99	9.1.E-01	0.99	8.9.E-01	HMDB0000086	Glycerophosphorylcholine
48	HMDB0002428	Terephthalate	0.97	1.2.E-01	0.97	4.2.E-02	0.99	5.8.E-01	0.99	7.2.E-01	0.96	1.7.E-01	0.96	2.6.E-01	1.01	9.4.E-01	1.01	9.0.E-01	HMDB0002428	Terephthalate
49	HMDB0004827	proline betaine	0.90	1.2.E-02	0.91	5.1.E-02	0.91	5.4.E-02	0.92	2.2.E-01	0.88	1.5.E-01	0.89	2.0.E-01	0.97	6.8.E-01	0.98	8.9.E-01	HMDB0004827	proline betaine
50	HMDB0000161	Ala	1.03	1.2.E-03	1.02	6.7.E-02	1.02	7.6.E-02	1.00	8.4.E-01	1.07	1.1.E-04	1.04	2.4.E-02	1.03	4.0.E-02	1.01	7.8.E-01	HMDB0000161	Ala
51	HMDB0000177	His	1.02	1.5.E-03	1.01	7.5.E-02	1.01	5.3.E-01	1.00	8.0.E-01	1.03	2.6.E-03	1.01	2.0.E-01	1.02	5.5.E-03	1.01	5.4.E-01	HMDB0000177	His
52	HMDB0000191	Asp	1.04	3.0.E-02	1.04	7.8.E-02	1.03	1.5.E-01	1.01	7.2.E-01	1.10	9.9.E-03	1.09	3.8.E-02	1.09	4.2.E-03	1.06	1.4.E-01	HMDB0000191	Asp
53	HMDB0000254	Succinate	0.99	2.1.E-01	0.98	8.9.E-02	0.99	1.9.E-01	0.99	5.7.E-01	0.99	7.1.E-01	1.00	9.1.E-01	1.00	9.4.E-01	1.00	9.0.E-01	HMDB0000254	Succinate
54	HMDB0001161	gamma-Butyrobetaine	0.97	3.2.E-03	0.98	9.2.E-02	0.98	1.6.E-01	0.99	5.4.E-01	0.99	7.8.E-01	1.01	7.1.E-01	0.97	1.1.E-02	0.98	3.4.E-01	HMDB0001161	gamma-Butyrobetaine
55	HMDB0000056	beta-Ala	0.96	1.5.E-03	0.98	1.0.E-01	0.99	4.2.E-01	0.99	4.7.E-01	1.01	7.8.E-01	1.01	8.6.E-01	1.04	1.2.E-01	1.03	3.9.E-01	HMDB0000056	beta-Ala

56	HMDB0000271	Sarcosine	1.07	2.3.E-02	1.05	1.0.E-01	1.00	9.3.E-01	0.99	8.4.E-01	1.11	5.6.E-02	1.11	7.9.E-02	0.99	9.1.E-01	0.99	8.9.E-01	HMDB0000271	Sarcosine
57	HMDB0001539	ADMA	1.05	7.9.E-02	1.05	1.0.E-01	1.02	5.8.E-01	1.02	5.7.E-01	1.12	3.9.E-02	1.11	7.6.E-02	1.03	4.9.E-01	1.04	6.0.E-01	HMDB0001539	ADMA
58	HMDB0000126	Glycerophosphate	0.98	5.5.E-01	0.96	1.0.E-01	1.03	2.9.E-01	1.00	9.3.E-01	0.93	7.2.E-02	0.91	2.3.E-02	1.01	9.4.E-01	1.01	9.0.E-01	HMDB0000126	Glycerophosphate
59	HMDB0000929	Trp	1.02	1.2.E-03	1.01	1.2.E-01	1.00	7.9.E-01	0.99	5.3.E-01	1.05	2.2.E-04	1.02	8.2.E-02	1.01	2.2.E-01	1.00	9.9.E-01	HMDB0000929	Trp
60	HMDB0000699	1-Methylnicotinamide	1.08	8.4.E-02	1.07	1.4.E-01	1.05	4.2.E-01	1.02	7.8.E-01	0.90	1.9.E-01	0.86	5.0.E-02	0.96	5.5.E-01	0.92	4.4.E-01	HMDB0000699	1-Methylnicotinamide
61	HMDB0000208	2-Oxoglutarate	1.00	9.4.E-01	0.98	1.7.E-01	1.03	7.3.E-02	1.01	7.5.E-01	0.99	7.1.E-01	0.99	7.5.E-01	1.02	7.7.E-01	1.01	9.0.E-01	HMDB0000208	2-Oxoglutarate
62	HMDB0000847	Pelargonate	1.02	2.3.E-01	1.02	1.7.E-01	1.01	4.5.E-01	1.01	5.7.E-01	1.00	1.0.E+00	1.01	7.7.E-01	1.01	9.4.E-01	1.01	9.0.E-01	HMDB0000847	Pelargonate
63	HMDB0000201	o-Acetylcarnitine	1.07	9.1.E-02	1.06	1.7.E-01	1.08	7.1.E-02	1.04	4.7.E-01	0.97	7.5.E-01	0.98	7.9.E-01	1.03	5.8.E-01	1.02	8.9.E-01	HMDB0000201	o-Acetylcarnitine
64	HMDB0000925	Trimethylamine N-oxide	0.94	1.8.E-01	0.94	1.9.E-01	0.96	4.6.E-01	0.95	4.7.E-01	1.03	7.5.E-01	1.01	8.8.E-01	1.12	4.1.E-02	1.09	3.9.E-01	HMDB0000925	Trimethylamine N-oxide
65	HMDB0000159	Phe	1.00	9.2.E-01	1.01	2.1.E-01	1.00	6.1.E-01	0.99	4.7.E-01	1.02	5.6.E-02	1.02	1.3.E-01	1.01	2.3.E-01	1.01	7.8.E-01	HMDB0000159	Phe
66	HMDB0000192	Cystine	0.98	1.2.E-02	0.99	2.1.E-01	1.00	5.8.E-01	1.00	6.5.E-01	1.00	8.0.E-01	0.99	6.7.E-01	1.00	8.3.E-01	0.99	5.9.E-01	HMDB0000192	Cystine
67	HMDB0000479	3-Methylhistidine	0.96	6.8.E-02	0.97	2.4.E-01	0.96	1.5.E-01	0.96	2.7.E-01	0.94	1.9.E-01	0.95	2.8.E-01	0.99	8.3.E-01	0.99	8.9.E-01	HMDB0000479	3-Methylhistidine
68	HMDB0000883	Val	1.00	8.6.E-01	1.01	2.4.E-01	1.00	7.0.E-01	0.99	4.7.E-01	1.02	8.7.E-02	1.01	2.7.E-01	1.01	2.2.E-01	1.00	8.9.E-01	HMDB0000883	Val
69	HMDB0000064	Creatine	1.01	7.9.E-01	0.97	2.4.E-01	0.99	7.0.E-01	0.96	1.8.E-01	0.99	7.2.E-01	0.98	4.8.E-01	0.96	8.5.E-02	0.94	4.2.E-02	HMDB0000064	Creatine
70	HMDB0000134	Fumarate	0.98	5.8.E-01	0.97	2.7.E-01	1.05	1.0.E-01	1.04	4.6.E-01	1.03	5.4.E-01	1.04	4.3.E-01	0.99	9.4.E-01	1.00	9.4.E-01	HMDB0000134	Fumarate
71	HMDB0000731	Cysteine S-sulfate	0.96	2.9.E-01	0.96	2.7.E-01	0.98	5.8.E-01	0.98	7.5.E-01	1.11	9.8.E-02	1.10	1.8.E-01	1.07	3.3.E-01	1.07	6.2.E-01	HMDB0000731	Cysteine S-sulfate
72	HMDB0000510	alpha-Aminoadipate	0.96	1.6.E-01	0.97	2.7.E-01	1.00	9.3.E-01	0.99	8.0.E-01	1.00	9.4.E-01	0.97	6.3.E-01	1.04	4.5.E-01	1.01	8.9.E-01	HMDB0000510	alpha-Aminoadipate
73	HMDB0000190	Lactate	1.03	2.2.E-02	1.01	2.9.E-01	1.03	2.6.E-02	1.01	7.2.E-01	1.04	1.3.E-01	1.04	1.6.E-01	1.00	9.4.E-01	1.00	9.0.E-01	HMDB0000190	Lactate
74	HMDB0000251	Taurine	0.99	4.2.E-01	0.99	3.0.E-01	0.99	5.8.E-01	1.00	8.0.E-01	1.01	5.1.E-01	1.00	8.4.E-01	1.01	3.8.E-01	1.01	5.9.E-01	HMDB0000251	Taurine
75	HMDB0000641	Gln	0.99	3.0.E-02	1.01	3.4.E-01	0.98	6.0.E-04	1.00	6.5.E-01	1.01	6.5.E-01	1.01	2.8.E-01	0.99	4.8.E-02	0.99	5.4.E-01	HMDB0000641	Gln
76	HMDB0032538	Triethanolamine	1.03	6.4.E-01	1.05	3.9.E-01	1.04	5.8.E-01	1.05	5.7.E-01	0.95	7.2.E-01	0.97	7.7.E-01	0.95	4.9.E-01	0.95	7.8.E-01	HMDB0032538	Triethanolamine
77	HMDB0003334	SDMA	0.97	2.1.E-01	0.98	4.4.E-01	0.98	5.8.E-01	0.99	8.0.E-01	0.93	1.9.E-01	0.92	1.7.E-01	1.00	9.7.E-01	1.00	9.9.E-01	HMDB0003334	SDMA
78	HMDB0000812	N-Acetylaspartate	0.99	6.3.E-01	0.99	4.9.E-01	0.99	7.1.E-01	0.99	7.8.E-01	1.01	8.7.E-01	1.01	7.1.E-01	1.01	9.4.E-01	1.02	9.0.E-01	HMDB0000812	N-Acetylaspartate
79	HMDB0000289	Urate	1.00	9.3.E-01	0.99	4.9.E-01	1.04	8.3.E-05	1.02	7.9.E-02	1.04	8.3.E-02	1.01	7.6.E-01	1.06	8.0.E-05	1.03	4.4.E-01	HMDB0000289	Urate
80	HMDB0000193	Isocitrate	1.01	4.0.E-01	1.01	5.7.E-01	1.04	2.1.E-03	1.02	2.6.E-01	1.00	9.7.E-01	1.01	7.6.E-01	1.02	4.3.E-01	1.02	7.6.E-01	HMDB0000193	Isocitrate
81	HMDB0061881	4-Acetylbutyrate	1.03	5.5.E-01	1.03	5.8.E-01	1.01	7.1.E-01	1.02	7.5.E-01	1.04	6.4.E-01	1.02	7.6.E-01	1.01	9.4.E-01	1.01	9.0.E-01	HMDB0061881	4-Acetylbutyrate
82	HMDB0000784	Azelate	1.01	7.5.E-01	1.01	5.8.E-01	1.01	7.4.E-01	1.01	7.5.E-01	1.03	3.7.E-01	1.03	5.3.E-01	1.00	9.4.E-01	1.01	9.0.E-01	HMDB0000784	Azelate
83	HMDB0000127	Glucuronate	1.00	9.1.E-01	1.02	5.8.E-01	1.02	5.0.E-01	1.01	7.5.E-01	1.06	2.5.E-01	1.05	4.3.E-01	1.03	7.7.E-01	1.02	9.0.E-01	HMDB0000127	Glucuronate
84	HMDB0000097	Choline	1.02	5.4.E-02	1.00	6.0.E-01	1.01	2.6.E-01	1.00	8.0.E-01	1.02	1.2.E-01	1.02	3.4.E-01	1.01	4.0.E-01	1.00	8.9.E-01	HMDB0000097	Choline
85	HMDB0000696	Met	1.00	7.9.E-01	1.01	6.4.E-01	0.99	5.8.E-01	0.99	5.1.E-01	1.04	8.7.E-02	1.03	2.2.E-01	1.01	6.9.E-01	1.00	8.9.E-01	HMDB0000696	Met
86	HMDB0000482	Octanoate	0.99	7.7.E-01	0.98	6.5.E-01	1.00	9.5.E-01	1.00	9.3.E-01	0.91	8.9.E-02	0.93	2.6.E-01	1.00	9.4.E-01	1.02	9.0.E-01	HMDB0000482	Octanoate
87	HMDB0000118	Homovanillate	1.05	3.1.E-01	1.02	6.8.E-01	1.09	3.5.E-02	1.06	3.3.E-01	1.03	7.1.E-01	1.00	9.8.E-01	1.00	9.9.E-01	0.98	9.0.E-01	HMDB0000118	Homovanillate
88	HMDB0000763	Indole-3-acetate	1.00	9.0.E-01	0.99	6.8.E-01	1.00	9.3.E-01	0.99	8.0.E-01	0.98	7.8.E-01	0.95	5.4.E-01	1.04	4.9.E-01	1.04	7.7.E-01	HMDB0000763	Indole-3-acetate
89	HMDB0000661	Glutarate	0.98	4.3.E-01	0.99	8.1.E-01	1.01	6.8.E-01	1.01	7.2.E-01	0.96	2.8.E-01	1.00	9.9.E-01	0.99	9.4.E-01	1.01	9.0.E-01	HMDB0000661	Glutarate
90	HMDB0000691	Malonate	1.00	9.6.E-01	0.99	8.2.E-01	0.99	8.8.E-01	0.99	8.5.E-01	1.05	5.2.E-01	1.05	5.3.E-01	1.01	9.4.E-01	1.02	9.0.E-01	HMDB0000691	Malonate
91	HMDB0000267	5-Oxoproline	0.99	4.3.E-01	1.00	8.2.E-01	0.99	3.2.E-01	1.00	9.3.E-01	1.03	5.7.E-02	1.04	2.0.E-02	1.01	7.7.E-01	1.02	7.6.E-01	HMDB0000267	5-Oxoproline
92	HMDB0000714	Hippurate	0.94	5.2.E-01	0.98	8.2.E-01	0.85	3.5.E-02	0.93	5.7.E-01	0.95	7.1.E-01	0.96	7.6.E-01	0.98	9.4.E-01	1.02	9.0.E-01	HMDB0000714	Hippurate
93	HMDB0000666	Heptanoate	0.97	4.3.E-01	0.99	8.3.E-01	0.97	4.5.E-01	0.98	7.5.E-01	0.99	8.8.E-01	0.97	7.6.E-01	0.94	5.3.E-01	0.93	6.2.E-01	HMDB0000666	Heptanoate
94	HMDB0000535	Hexanoate	0.99	6.4.E-01	1.00	8.7.E-01	0.98	2.1.E-01	0.98	5.7.E-01	0.93	7.1.E-03	0.93	2.0.E-02	0.99	9.4.E-01	0.99	9.0.E-01	HMDB0000535	Hexanoate

FDR, false discovery rate.

eTable 2. Characteristics of the participants at the follow-up survey after matching

	HTP users	Cigarette smokers	Past smokers	Never smokers
N, %	55 (11.9)	119 (25.7)	144 (31.1)	145 (31.3)
Women, n (%)	10 (18.2)	27 (22.7)	30 (20.7)	30 (20.7)
Age, mean (SD)	49.22 (10.57)	54.51 (8.83)	52.72 (10.25)	51.34 (10.08)
Total tobacco products number per day	13.12 (4.82)	14.41 (6.23)		
Cigarette, mean (SD)		14.41 (6.23)		
IQOS, mean (SD)	11.05 (6.41)			
Ploom TECH, mean (SD)	0.27 (2.02)			
glo, mean (SD)	1.80 (5.06)			
Years of using HTPs	1.69 (0.84)			
Cigarette number per day at baseline	15.49 (6.14)	16.08 (6.80)		
Years of smoking	27.47 (10.94)	33.00 (10.23)	23.25 (15.10)	
Years after cessation			8.77 (7.65)	
Daily drinking, %	42 (76.4)	90 (75.6)	104 (72.2)	108 (74.5)
Systolic blood pressure, mean (SD)	128.93 (17.56)	129.93 (17.99)	131.51 (16.44)	128.61 (15.80)
BMI, mean (SD)	23.00 (3.03)	23.05 (3.75)	23.77 (3.66)	23.86 (3.61)
HbA1c, mean (SD)	5.66 (0.60)	5.71 (0.47)	5.75 (0.59)	5.66 (0.56)
Triglyceride, median (IQR]	94.0 (58.0–134.5)	108.0 (79.5–146.5)	92.0 (65.8–144.3)	95.0 (62.0–132.0)

HTP, heated tobacco products; IQR, interquartile range; SD, standard deviation.

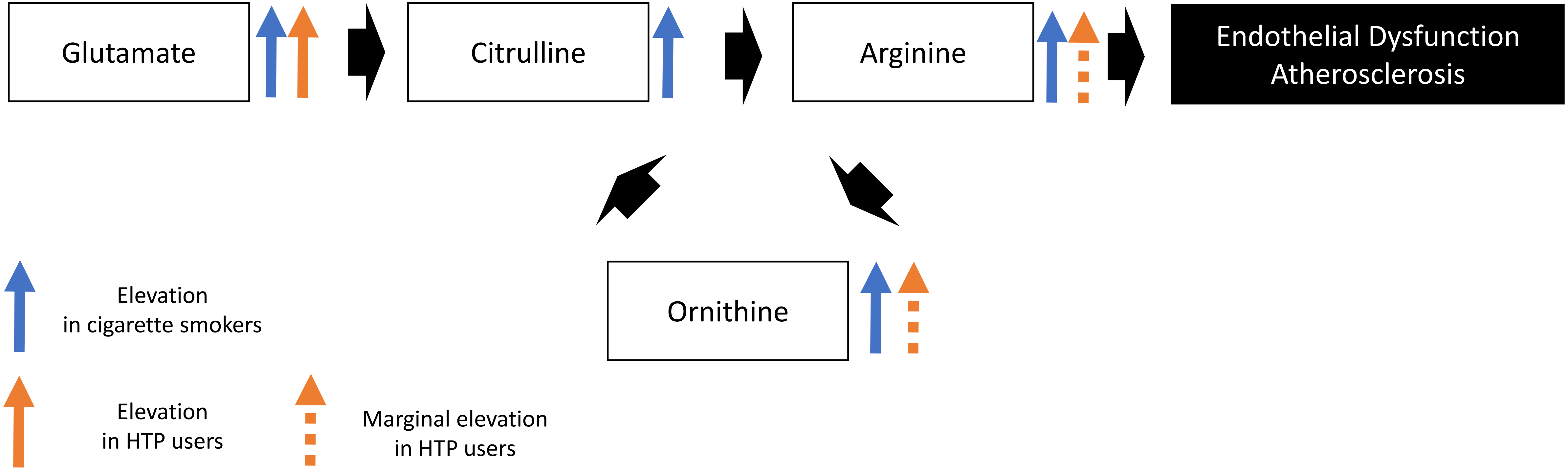

eFigure 1. Glutamate pathway and atherosclerosis development in smokers.

Previous studies reported that the glutamate transporter encoded by SLC7A11 was activated in cigarette smokers, which increased circulating glutamate, arginine, ornithine, and citrulline.[23,24] In our study, the elevation of these metabolites was confirmed. Previous studies have suggested that elevation of this metabolic pathway can be linked to endothelial cell damage through nitric oxide decline and contribute to atherosclerosis.[25-27]
